# Interval exercise increases angiogenic cell function in postmenopausal women

**DOI:** 10.1136/bmjsem-2017-000248

**Published:** 2017-08-06

**Authors:** Emma Harris, Mark Rakobowchuk, Karen M Birch

**Affiliations:** 1 School of Human and Health Sciences, University of Huddersfield, Huddersfield, UK; 2 Department of Biological Sciences, Faculty of Science, Thompson Rivers University, Kamloops, British Columbia, Canada; 3 Multidisciplinary Cardiovascular Research Centre, Faculty of Biological Sciences, University of Leeds, Leeds, UK

**Keywords:** menopause, interval and continuous exercise, endothelial function, circulating angiogenic cells

## Abstract

**Introduction:**

Exercise can help to negate the increased cardiovascular disease risk observed in women after the menopausal transition. This study sought to determine whether interval or continuous exercise has differential effects on endothelial function and circulating angiogenic cell (CAC) number and function in postmenopausal women.

**Methods:**

Fifteen healthy postmenopausal women completed a 30 min acute moderate-intensity continuous (CON) and interval exercise (MOD-INT) session on a cycle ergometer on separate days. Nine participants completed a further single 30 min acute heavy-intensity interval (HEAVY-INT) exercise session. Brachial artery flow-mediated dilation (FMD) was assessed pre-exercise and 15 min post-exercise session. CAC number and colony-forming capacity in vitro were assessed post exercise and compared with resting levels.

**Results:**

FMD and CAC number did not change post exercise regardless of exercise type (p>0.05). However, the number (mean±SD) of colony-forming units (CFUs) increased from visit 1 (12±10 CFUs/well) to post MOD-INT (32±30 CFUs/well) and post HEAVY-INT (38±23 CFUs/well) but not post CON (13±14 CFUs/well).

**Conclusion:**

A single session of interval exercise is more effective than a continuous exercise session for increasing the intercellular communication of CACs, regardless of exercise intensity. The enhanced ability of CACs to form colonies may reflect an increased number and/or function of angiogenic T-cells. The repeated exertions to higher work rates during interval exercise may explain this response. Repeated exercise sessions might be required to improve FMD in postmenopausal women.

**What are the new findings?**

- An acute bout of interval exercise increases the ability of circulating angiogenic cells to form colonies, which may increase the capacity for vascular repair in postmenopausal women.
- The government-recommended guidelines of 30 min of moderate-intensity continuous exercise did not acutely impact endothelial function or factors associated with repair in postmenopausal women.

**How might it impact on clinical practice in the near future?**

- The findings provide support for individualised exercise prescription for postmenopausal women.
- Healthcare professionals may advise postmenopausal women to exercise in short bursts of activity followed by active rest periods for vascular benefits.

## Introduction

The risk of cardiovascular disease (CVD) in women increases during perimenopause and postmenopause primarily due to the loss of oestrogen. This hormonal change, in addition to increasing age, reduces endothelial function^1^ and the number and function of circulating angiogenic cells (CACs; ^15^). CACs migrate and aggregate to areas of vascular damage and aid in endothelial repair through the secretion of proangiogenic cytokines and growth factors.^2^ Low levels of CACs in circulation and reduced function following culture (ie, impaired colony-forming capacity) are associated with an increased risk of developing CVD.^3^ Fewer numbers of colony-forming units (CFUs) are associated with poorer brachial artery endothelial function and increased severity of coronary artery disease.^4 5^ Decreased oestrogen levels significantly impact the capacity for endothelial repair.^6^ Thus, the reduction in endothelial function and inability of CACs to aid in vascular repair following the menopausal transition indicates an imbalance in vascular homeostasis and plays a part in CVD risk.

Lifestyle interventions such as exercise are recommended for postmenopausal women as a preventative strategy for CVD.^7^ Aerobic exercise training studies in postmenopausal women have demonstrated improved endothelial function^8^ and reduced oxidative stress.^9^ However, the effect of exercise on CAC number and function in postmenopausal women has not been studied. Recent evidence suggests that interval exercise is more or equally effective as UK government guidelines for moderate-intensity continuous exercise in improving cardiorespiratory fitness, endothelial function and arterial stiffness.^10 11^ We have suggested that interval exercise performed at a heavy intensity may induce an increase in CAC number compared with moderate-intensity exercise, likely due to greater metabolic stress.^2 12^ However, a comparison between the effects of interval and continuous exercise on endothelial function, CAC number and function of these cells has not been examined in postmenopausal women.

The aims of this study were to (1) compare the acute effects of moderate-intensity continuous and interval exercise, on endothelial function and CAC number/function in postmenopausal women, and (2) compare these effects with an interval exercise session performed in the heavy-intensity domain. Endothelial function measured by brachial artery flow-mediated dilation (FMD) was the primary outcome. Secondary outcomes were brachial artery diameter, shear rate and reactive hyperaemic variables and CAC number and function. We did not include a session of heavy-intensity continuous exercise as reports suggest that postmenopausal women dislike exercise of this nature.^13^ Investigating the acute effects of exercise on vascular health allows identification of (1) exercise that has an immediate impact and (2) the type and intensity of exercise that may yield the greatest improvements if undertaken chronically. We hypothesised that interval exercise would be tolerable and would improve markers of vascular health and repair to a greater extent than continuous exercise due to the brief excursions to higher work rates.

## Methods

### Participants

Fifteen healthy postmenopausal women (age: 63±4 years) volunteered for the study and provided written informed consent. Postmenopausal status was defined as absence of a menstrual cycle for >2 years and confirmed through follicle stimulating hormone >30 iU/L. Exclusion criteria included smoking, known cardiovascular, pulmonary and metabolic disease, musculoskeletal impairments, cancer, contradictions to exercise, medication use (eg, hormone replacement therapy) and if participants had given blood in the previous three months. Participants were not currently exercising more than twice per week. Ethical approval was provided by the University of Leeds Faculty of Biological Sciences Ethics Committee and all procedures conformed to the Declaration of Helsinki.

### Experimental protocol

For the assessment of endothelial function and calculation of work rate for the exercise bouts, participants attended the laboratory on two occasions (separated by 1 week). Participants were instructed to refrain from exercise participation and consuming alcohol and caffeine for 12 hours prior to each visit. At the first visit, a fasted blood sample (~50 mL) for the assessment of CVD risk blood markers and CAC number and function was completed. At the second visit, a cardiorespiratory fitness test was completed to determine peak aerobic capacity ($\dot{V}O_{2peak}$) and lactate threshold. On three further occasions, each separated by ≥1 week (to allow exercise-induced changes in CACs to return to baseline levels), participants completed 30 min of either moderate-intensity continuous (CON), moderate-intensity interval (MOD-INT) or heavy-intensity interval (HEAVY-INT) exercise bouts. Only nine participants completed the heavy-intensity bout. To assess the acute effects of each exercise bout on endothelial function, assessments were completed before and 15 min after exercise. For CAC number and function, blood samples were acquired 30 min post exercise and results compared with that of visit 1.

### Variables assessed before and after exercise sessions

#### Cardiorespiratory fitness

A seated ramp-incremental exercise test (10 W/min) was performed for the assessment of $\dot{V}O_{2peak}$ and the lactate threshold, and for calculation of the work rates achieved at these points. These values were used to establish work rates for the subsequent exercise sessions. Participants were seated on an electronically braked cycle ergometer (Excalibur Sport V.2.0; Lode BV, Groningen, The Netherlands) and a mouthpiece and nose clip were fitted for breath by breath analysis of pulmonary gas exchange. The protocol has been described in detail previously.^12^ Heart rate, blood pressure and rating of perceived exertion (Borg's scale of rating of perceived exertion) were measured every 2 min during the test using a 12-lead ECG, sphygmomanometer and a visual scale of exertion (6-20), respectively. The work rate at the end of the ramp-incremental test was calculated as test duration × ramp rate (10)+20W. Breath-by-breath data were exported and analysed using OriginPro software (OriginPro 8, OriginLab, Northampton, Massachusetts, USA). Breaths were eliminated if $\dot{V}O_{2peak}$ values fell outside 4 SD from the local mean. As previously described,^24^ a 12-breath moving average was calculated and the highest value was defined as $\dot{V}O_{2peak}$. The estimated lactate threshold was determined using the V-slope method^25^ and confirmed by a rise in end-tidal O_2_ and plateau in end-tidal CO_2_.

#### Endothelial function

Endothelial function was assessed in the morning in a temperature-controlled laboratory (20–24°C) after 20 min supine rest. The protocols for our lab have been described in detail elsewhere.^2 12^ Briefly, endothelial function was assessed by brachial artery FMD using ultrasound imaging following a 5 min period of forearm occlusion. Images were recorded at end-diastole using vascular imaging software (Vascular Imager, Medical Imaging Applications, Coralville, Iowa, USA) and analysed using semiautomated edge-detection software (Brachial Tools V.5, Medical Imaging Applications) to determine brachial artery diameter. Peak reactive hyperaemia, peak shear rate, area under the shear rate curve from cuff release to peak dilation (AUC_peak_) and to 60 s (AUC_60_) and 90 s (AUC_90_) post cuff release, and their corresponding velocity time integrals (VTIs) were also calculated. FMD was not normalised to shear rate/AUC as not all assumptions for the use of ratios were met.^26^ During recording of blood velocity, the insonation angle for each participant before and after exercise and between different exercise visits was within 2°. Day-to-day coefficient of variation for FMD in our lab is 15%.^2^

#### Blood markers and CAC number and function

Visit 1 blood samples were analysed by local hospital pathology services for serum follicle stimulating hormone levels, lipoproteins, insulin, glucose and HbA1c. CACs were enumerated from 22 mL of blood via flow cytometry using a commercially available kit (Miltenyi Biotec) as previously described.^2^ CACs were defined as CD34^+^, double positive (CD34^+^KDR^+^) or triple positive (CD34^+^KDR^+^CD133^+^). To assess the in vitro function of CACs, a CFU assay was performed, according to the manufacturer's instructions. Briefly, peripheral blood mononuclear cells were separated by Ficoll density-gradient centrifugation of whole blood (Ficoll Paque PLUS, GE Healthcare, Buckinghamshire, UK), and 5×10^6^ cells were suspended in 2 mL of EndoCult growth medium (StemCell Technologies, Vancouver, Canada) and cultured in one well of a fibronectin-coated six-well plate for 48 hours at 37°C in 5% CO_2_. After 48 hours, the non-adherent cells were collected from the well and seeded in duplicate on a 24-well fibronectin-coated plate at a density of 1×10^6^ cells/well. Following three further days of culture, the non-adherent cells were removed and the number of CFUs per well counted and an average calculated. CFUs were defined as clusters of >100 round cells with spindle-shaped cells surrounding the core.

#### Exercise session protocols

Participants completed 30 min CON, MOD-INT and HEAVY-INT exercise bouts on a cycle ergometer (Excalibur Sport V.2.0) on separate days. Duration of 30 min was chosen as it reflects the current UK Department of Health guidelines of 30 min of moderate-intensity exercise, 5 days/week (ref. 27; p. 34). CON exercise involved cycling at 80% of the work rate achieved at the lactate threshold (moderate-intensity domain as $\dot{V}O_{2peak}$ remained below the lactate threshold^28^). The interval exercise bouts were based on work by Turner *et al*.^29^ Thus, our participants cycled at 90% of the work rate achieved at $\dot{V}O_{2peak}$ for duty cycles of 10:20 s for MOD-INT and 30:60 s for HEAVY-INT exercise. Active recovery was conducted at 10 W and confirmation of intensity domain was confirmed by stabilisation of oxygen uptake above or below the lactate threshold accordingly. By design the MOD-INT and HEAVY-INT exercise bouts were matched for average work rate and work completed.

#### Statistical analysis

All analyses was completed using SPSS V.22. Data were assessed for normal distribution via Kolmogorov-Smirnov. A non-parametric Friedman's analysis of variance (ANOVA) was conducted on CAC number only as data were not normally distributed and could not be transformed. The remaining data were examined by a mixed-design ANOVA with time (pre vs post exercise) as the within-subject factor and exercise bout (CON, MOD-INT and HEAVY-INT) as the between-subject factor. Pearson's correlations were performed between $\dot{V}O_{2peak}$, blood pressure, full lipid profile and the change in CFUs post exercise. Using previously reported acute increases of 4.6% in FMD following 45 min of treadmill exercise in postmenopausal women,^17^ and SD of 3% calculated from our lab,^12^ a minimum of nine participants in total were required to obtain 80% power (α=0.05) in a crossover study. Data are presented as mean±SD and percentage change with accompanying 95% CI.

## Results

### Participant and exercise session characteristics

Participant characteristics are displayed in table 1. Total cholesterol and low-density lipoprotein levels were higher than the desirable healthy range (>5.2 and >3.4 mmol/L, respectively) in nine and seven participants, respectively. By design, work completed (54±3 kJ) during MOD-INT and HEAVY-INT exercise sessions was equal. The average work rate of the peaks during MOD-INT and HEAVY-INT (70±5 W) were significantly higher than the average work rate of CON exercise (35±6 W).

**Table 1:** Participant characteristics of postmenopausal women at visit 1

	n	Mean (±SD)
Age (years)	15	63±4
BMI (kg/m)	15	25±3
Brachial artery SBP (mm Hg)	15	137±15
Brachial artery DBP (mm Hg)	15	84±5
Brachial artery MAP (mm Hg)	15	102±8
Absolute $\dot{V}O_{2peak}$ (L/min)	15	1.40±0.29
Relative $\dot{V}O_{2peak}$ (mL/kg/min)	15	21.6±5.4
Plasma glucose (mmol/L)	13	4.8±0.5
HbA1c (mmol/mol/HB)	14	39±2
Total cholesterol (mmol/L)	14	5.9±1.0
HDL (mmol/L)	14	1.9±0.5
LDL (mmol/L)	14	3.5±0.8
Cholesterol:HDL ratio	14	3.1±0.7
Triglycerides (mmol/L)	14	1.0±0.4
FSH (iU/L)	13	69.9±31.0
Insulin (mU/L)	14	6.5±3.1

BMI, body mass index; DBP, diastolic blood pressure; FSH, follicle-stimulating hormone; HbA1c, haemoglobin A1c; HDL, high-density lipoprotein; LDL, low-density lipoprotein; MAP, mean arterial pressure; SBP, systolic blood pressure.

### Brachial artery endothelial function

Brachial artery endothelial function was unaltered by acute CON, MOD-INT and HEAVY-INT exercise (p>0.05, table 2). There were no significant post-exercise changes in brachial artery resting diameter (p=0.72) or the associated shear rate and reactive hyperaemic variables (p>0.05). Additionally, there were no time by exercise interactions (p>0.05).

**Table 2:** Brachial artery endothelial function (mean±SD) pre and post an acute 30 min bout of moderate-intensity continuous (CON, n=15), moderate-intensity interval (MOD-INT, n=14) and heavy-intensity interval (HEAVY-INT) exercise (n=9)

	CON		MOD-INT		HEAVY-INT	
	Pre	Post	Pre	Post	Pre	Post
Resting diameter (mm)	3.4±0.5	3.4±0.4	3.5±0.4	3.4±0.4	3.2±0.5	3.2±0.5
Time from cuff release to peak diameter (s)	45±17	49±23	53±16	51±19	69±20	75±27
Insonation angle (°)	68±1	68±1	68±1	68±1	68±1	68±1
Relative FMD (%)	6.1±2.5	6.0±4.2	5.3±2.5	5.3±3.2	4.9±1.7	4.3±1.7
VTI_peak_ (cm)	1442±602	1620±775	1681±597	1719±709	2198±526	2213±835
VTI_60_ (cm)	1634±5493	1797±565	1806±435	1861±410	2106±472	1909±455
VTI_90_ (cm)	2111±648	2261±765	2281±564	2364±579	2550±650	2368±592
Peak reactive hyperaemia (cm/s)	71±28	74±32	88±33	87±25	110±14	104±23
Peak shear rate (/s^–1^)	1635±659	1746±813	2072±892	2096±723	2830±557	2640±573
AUC_peak_ (a.u.)	33 204±15 071	37 500±16 200	39 477±15 399	40 027±14 399	55 508±13 056	55 647±19 664
AUC_60_ (a.u.)	37 825±12 899	42 027±12 830	42 292±11 723	44 053±9443	53 132±11 319	48 403±12 482
AUC_90_ (a.u.)	48 785±16 752	52 657±16 587	53 211±14 127	55 796±12 521	64 237±15 371	59 953±16 039

No significant effects of exercise or group by time interactions were revealed (p>0.05).

AUC, shear rate area under the curve; FMD, flow-mediated dilation; VTI, velocity–time integral.

### CAC number

Neither CON nor MOD-INT exercise altered concentrations of CD34^+^ (p=0.28), CD34^+^KDR^+^ (p=0.57) or CD34^+^KDR^+^CD133^+^ cells (p=0.74). In addition, there was no change in any CAC population following HEAVY-INT (p>0.05, table 3). No time by exercise bout interactions were observed (p>0.05).

**Table 3:** Circulating angiogenic cell number (mean±SD) at visit 1 and post an acute 30 min bout of moderate-intensity continuous (CON) and interval (MOD-INT) exercise and heavy-intensity interval (HEAVY-INT) exercise

	Visit 1	CON	MOD-INT	HEAVY-INT
CD34^+^ cells /mL blood	2.7±1.3×10^5^	2.2±1.2×10^5^	2.6±1.1×10^5^	2.3±0.9×10^5^
CD34^+^KDR^+^ cells / mL blood	251±176	108±119	244±188	144±201
CD34^+^KDR^+^CD133^+^ cells /mL blood	68±102	11±13	40±85	14±26

No significant effects of exercise or group by time interactions were revealed (p>0.05).

### CFU numbers

**Figure 1:**
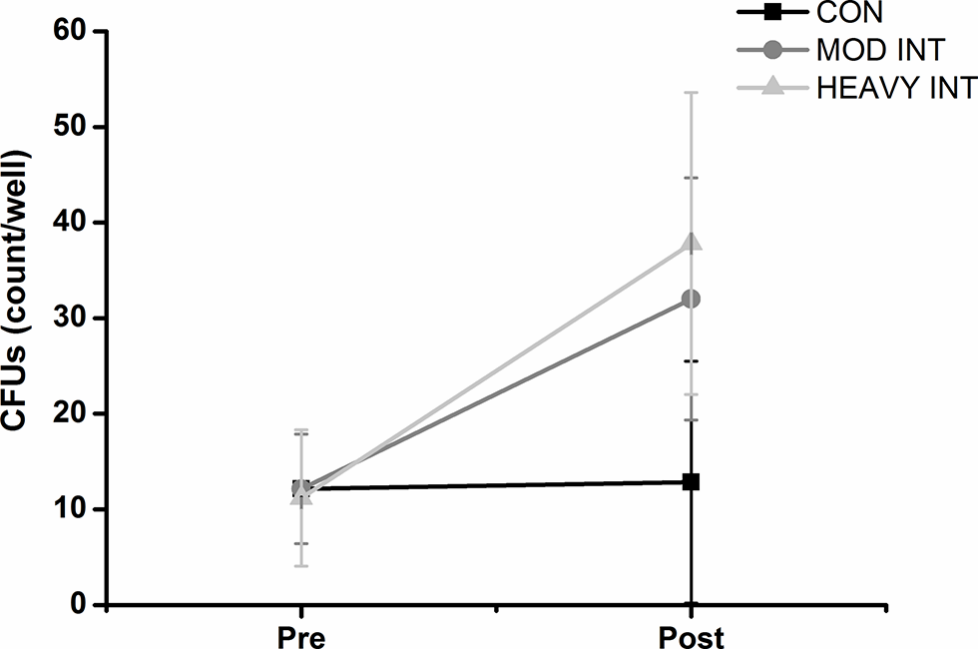
The number (mean with 95% CI) of colony-forming units (CFUs) at visit 1 and following a 30 min moderate-intensity continuous (CON), moderate-intensity interval (MOD-INT) and heavy-intensity interval (HEAVY-INT) exercise bout. CFUs increased following the MOD-INT (n=14) and the HEAVY-INT (n=9) exercise bouts, but not following CON (n=14).

A significant time effect (p=0.0001) and time by exercise bout interaction (p=0.017) for CFU number was revealed. The analysis of the means and 95% CI for pre exercise and post exercise (figure 1) illustrates that CFU number increased following MOD-INT (pre: 12.2 CFUs, 95% CI 6.4 to 17.9; post: 32 CFUs, 95% CI 19.4 to 44.7) and HEAVY-INT (pre: 11.2 CFUs, 95% CI 4.1 to 18.3; post: 37.8 CFUs, 95% CI 22 to 53.6) but remained unchanged following CON (pre: 12.2 CFUs, 95% CI 6.4 to 17.9; post: 12.8 CFUs, 95% CI 0.2 to 25.5). There was no correlation between the change in CFUs and pre-exercise  $\dot{V}O_{2peak}$, blood pressure or cholesterol levels (p>0.05).

## Discussion

The present study was the first to compare the acute effects of continuous and interval exercise on endothelial function and CAC number and function in postmenopausal women. The population of postmenopausal women studied were healthy but presented risk factors for CVD. The main findings were that moderate-intensity continuous exercise had no immediate effect on endothelial function or CAC concentrations or function. Conversely, MOD-INT and HEAVY-INT exercise acutely increased the ability of cultured CACs to form colonies in vitro. Presentation of CVD risk factors did not influence this effect.

### Continuous and interval exercise did not acutely effect endothelial function

Brachial artery endothelial function did not change following any of the exercise bouts. In response to lower-limb exercise, measurement of upper limb vascular function is used to reflect systemic endothelial function. Our findings suggest that for increases in systemic endothelial function in postmenopausal women to occur, a greater stimulus or repeated acute exercise bouts are required. Indeed, an absolute increase of ~5% in brachial artery FMD was observed by Harvey *et al.* following 45 min of continuous treadmill exercise at 60% $\dot{V}O_{2max},$ in postmenopausal women with similar FMD values as in the present study (~5%; ^17^). Greater endothelial function following exercise is mediated by shear stress-induced increases in nitric oxide bioavailability which induces vasodilation.^21^ The exercise duration was greater in Harvey's study and a different definition of intensity adopted compared with the present study. It is likely that a greater volume/magnitude of shear was thus induced.

HEAVY-INT exercise may well have been expected to induce higher levels of shear stress; however, compared with the women studied by Harvey *et al.*, women in the present study were older (64±4 years vs 54±2 years) and had greater blood pressure (137/84 mm Hg vs 108/64 mm Hg). Hypertension and older age (>60 years) have an additive effect on reducing nitric oxide bioavailability and increasing oxidative stress.^30^ Additionally, the sensitivity of the endothelium to detect shear stress and trigger nitric oxide synthesis may be reduced. Aged endothelial cells in vitro exhibit impaired eNOS protein upregulation in response to shear stress compared with young cells cultured under identical conditions.^19^ Thus, a greater shear stress stimulus may be required to induce increases in nitric oxide in the present cohort.

### Circulating angiogenic cells were not mobilised following exercise

The present study is the first to measure CAC mobilisation following exercise in postmenopausal women and use novel comparisons between interval and continuous exercise sessions. Although some studies have observed acute increases in CAC concentrations following maximal and submaximal exercise in healthy and diseased populations,^14 31 32^ others report no change.^33 34^ We observed no increase in CACs following continuous and interval exercise at moderate and heavy intensities. These discrepancies might be explained by differing populations, definitions of CACs, exercise stimuli and the techniques used to numerate cells. However, acute exercise-induced CAC mobilisation is mechanistically driven by an increase in shear stress-induced nitric oxide.^14^ Thus, if endothelial sensitivity is impaired in postmenopausal women, it may well be that a larger magnitude and/or volume of shear stress are required to mobilise CACs. This could be achieved by exercising at greater work rates or by repeated acute bouts in a training programme.

### Interval exercise acutely increases circulating angiogenic cell functional capacity

A low capacity of cultured CACs to form colonies is associated with a greater risk of CVD.^4^ The increase in CFUs following interval exercise regardless of intensity, but not following continuous exercise, suggests that interval exercise is more effective at increasing the capacity for endothelial repair by CACs among postmenopausal women. However, the mechanisms involved are unclear.

In vitro characterisation studies have demonstrated that CFUs are a heterogeneous population of aggregated monocytes and T cells^35^ and the assay reflects the intercellular communication of these cell populations.^18^ The ability to form CFUs depends on angiogenic T cells (located in the centre of the CFU colonies) that express the platelet endothelial cell adhesion molecule (CD31) and the stromal-derived factor-1 receptor (CXCR4).^20 36^ Higher levels of circulating angiogenic T cells are associated with more CFUs in vitro, even when equal numbers of peripheral blood mononuclear cells are cultured,^20^ while lower levels are associated with older age and increased CVD risk.^20 36^ Angiogenic T cells are mobilised immediately post exercise, and thus, it may be that interval exercise mobilised circulating angiogenic T cells to a greater extent than continuous exercise, thus, enabling more CFUs to form in vitro. Future studies should measure whether more angiogenic T cells (CD3^+^CD31^+^CXCR4^+^) are present post exercise.

Angiogenic T cells in culture may also enable CACs to form colonies through the secretion of proangiogenic cytokines.^20^ Messenger RNA expression of genes involved in immune and T cell function (eg, interleukin (IL)-1β and IL-8) are upregulated following acute cycling exercise.^37^ It is plausible that interval but not continuous exercise induced a change in this gene expression. Future studies should characterise the phenotype and gene expression of CFUs post exercise.

The mechanisms for increased CFUs post-interval exercise but not continuous exercise remain to be fully elucidated. Interval exercise induces greater peak heart rates and fluctuations in vascular shear rate profiles compared with moderate-intensity continuous exercise.^11 12 38^ Given that differentiation of peripheral blood mononuclear cells into different T cell subtypes is dependent on cytokine and catecholamine (ie, epinephrine, norepinephrine and cortisol) concentrations,^23 37 39^ the repeated fluctuations to higher work rates during interval exercise may act as a greater stimulus for angiogenic T cell mobilisation and activation essential for CFUs in vitro. Indeed, a recent study demonstrated that highly differentiated T cells were increased to a greater extent following interval exercise compared with continuous exercise, and regulatory T cells were only mobilised following interval exercise, likely due to increased plasma epinephrine.^22^

## Strengths and limitations

A strength of this study is the cross-over design which reduces between-participant variability as each participant acts as their own control. We recognise that the inclusion of a non-exercise control group would have further supported the conclusion that acute interval exercise increases CFU number. However, given that 30 min of continuous exercise did not alter CFU number we are confident using this as our reference group. Additionally, the mean insonation angle of 68° may overestimate blood velocity^26^; however, we reduced this impact by maintaining the same angle before and after exercise for each participant. It is important to note that this study only investigated the effects of single acute bouts of exercise on endothelial function and CAC number/function in postmenopausal women; different effects might be observed following repeated exercise sessions over longer time periods.

## Conclusions and future work

The UK government recommended guidelines of 30 min of moderate-intensity continuous exercise do not have an immediate impact on endothelial function and CAC number and function in postmenopausal women. In contrast, while MOD-INT or HEAVY-INT exercise did not increase the mobilisation of CACs, it did increase the colony-forming ability of peripheral blood mononuclear cells potentially involved in the repair of vascular damage. The potential impact of interval exercise for vascular health and repair in postmenopausal women and other populations that are at risk of developing CVD is significant. Studies manipulating the magnitudes and fluctuations in shear are now imperative.
